# Two critical periods in early visual cortex during figure–ground segregation

**DOI:** 10.1002/brb3.91

**Published:** 2012-09-29

**Authors:** Martijn E Wokke, Ilja G Sligte, H Steven Scholte, Victor A F Lamme

**Affiliations:** 1 Cognitive Neuroscience Group, Department of Psychology, University of AmsterdamWeesperplein 4, 1018 XA, Amsterdam, The Netherlands; 2 Cognitive Science Center, University of AmsterdamSarphatistraat 104, 1018 GV, Amsterdam, The Netherlands

**Keywords:** EEG, scene segmentation, TMS, V1/V2, visual perception

## Abstract

The ability to distinguish a figure from its background is crucial for visual perception. To date, it remains unresolved where and how in the visual system different stages of figure–ground segregation emerge. Neural correlates of figure border detection have consistently been found in early visual cortex (V1/V2). However, areas V1/V2 have also been frequently associated with later stages of figure–ground segregation (such as border ownership or surface segregation). To causally link activity in early visual cortex to different stages of figure–ground segregation, we briefly disrupted activity in areas V1/V2 at various moments in time using transcranial magnetic stimulation (TMS). Prior to stimulation we presented stimuli that made it possible to differentiate between figure border detection and surface segregation. We concurrently recorded electroencephalographic (EEG) signals to examine how neural correlates of figure–ground segregation were affected by TMS. Results show that disruption of V1/V2 in an early time window (96–119 msec) affected detection of figure stimuli and affected neural correlates of figure border detection, border ownership, and surface segregation. TMS applied in a relatively late time window (236–259 msec) selectively deteriorated performance associated with surface segregation. We conclude that areas V1/V2 are not only essential in an early stage of figure–ground segregation when figure borders are detected, but subsequently causally contribute to more sophisticated stages of figure–ground segregation such as surface segregation.

## Introduction

Fundamental for visual perception is the segregation of a scene into figure and background. In the process of figure–ground segregation, different stages can be discerned: an early stage in which figure borders are detected and a later stage when processes such as surface segregation and border ownership coding emerge (Lamme 1995; Zhou et al. 2000). For a long time, figure–ground segregation was thought to operate in a strictly hierarchical fashion. In the first stages of visual processing, small receptive fields in the primary visual cortex process elementary features (such as local contrasts, orientation, direction of motion [Livingstone and Hubel 1988; Zipser et al. 1996]), which serves as input for higher tier cortical regions. As information progresses upstream through the cortical hierarchy, receptive fields increase in size and their characteristics become more complex (Maunsell and Newsome 1987), allowing initially distributed information to become integrated (often referred to as “binding”). However, a growing number of studies demonstrate that areas V1/V2 are not only active in early stages of visual processing, when figure border detection takes place, but are also involved in the processing of higher level and context-dependent information (Zipser et al. 1996; Zhou et al. 2000; Albright and Stoner 2002; Juan and Walsh 2003; Fahrenfort et al. 2007). Contextual modulation of activity in V1/V2 arises when neurons in these areas increase or decrease their signaling based on information far beyond their classical receptive fields (cRF). For instance, contextual modulation in early visual cortex (V1/V2) is found when the cRF of a neuron covers a small part of the visual field belonging to a figure surface instead of being part of the background (surface segregation [Zipser et al. 1996]) or by the location of the figure with respect to the cRF (border ownership coding [Zhou et al. 2000]). In both examples, the cRF size is too small for the neuron to “know” whether it is inside a figure or to “see” on which side of the cRF a figure is located. Contextual modulation of signals in V1/V2 therefore seems to reflect integration of information over larger parts of the visual field.

Figure–ground manipulations have also been shown to influence relatively late (peri-occipital) event-related potential (ERP) components in human electroencephalographic (EEG) recordings (Lamme et al. 1992; Bach and Meigen 1997; Caputo and Casco 1999; Scholte et al. 2008; Pitts et al. 2011). These studies show an early effect related to figure border detection and a later occurring enhancement of activity likely reflecting border ownership coding and/or surface segregation.

Although figure–ground modulation of signals in V1/V2 is intriguing, it could be that these modulations are epiphenomenal, reflecting attention, some sort of by-product of activity higher upstream or residual lingering of local activity. In addition, the neural pathway mediating these modulations has been subject to debate for many years now (Kastner et al. 2000; Lamme and Spekreijse 2000; Rossi et al. 2001; Scholte et al. 2008; Supèr et al. 2010; Zhang and von der Heydt 2010).

To study the *necessity* of V1/V2 during different stages of figure–ground segregation, we disrupted activity in V1/V2 with transcranial magnetic stimulation (TMS) at different time intervals while concurrently recording EEG signals. We presented stimuli that made it possible to differentiate between figure border detection and surface segregation (Scholte 2003; Heinen et al. 2005; Scholte et al. 2008; Vandenbroucke et al. 2008). By combining TMS and EEG, we were able to determine how magnetic stimulation of V1/V2 affects neural signaling in early visual cortex over time and test how this neural activity causally relates to different stages in figure–ground segregation.

## Materials and Methods

### Participants

Fifteen undergraduate psychology students of the University of Amsterdam (14 females, mean age = 21.3, SD = 1.71) participated in this study for financial compensations (13 subjects participated in the TMS–EEG experiment, and two subjects participated exclusively in the TMS pilot [see “TMS protocol”]). All participants had normal or corrected-to-normal vision and were naïve to the purpose of the experiment. Participants had no history of neurological diseases or other risk factors and were screened prior to the experiment according to international guidelines (Wassermann 1998; Rossi et al. 2009). All procedures were approved by the Ethics Committee of the Psychology Department of the University of Amsterdam, and subjects gave their written informed consent prior to the experiment.

### Task design

**Figure 1:**
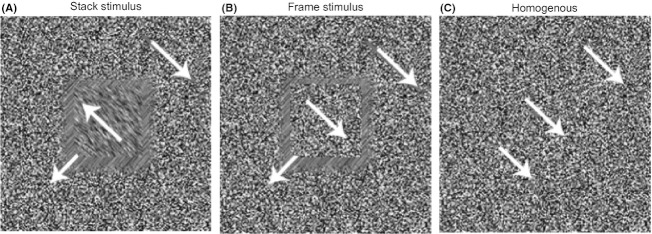
(A–C) Stimuli were created by displacing randomly distributed black-and-white dots in one of the four directions. The three stimuli differed in the amount of figure regions segregated from the background. Animated versions of the stimuli are visible by clicking on the stimulus.

**Figure 2:**
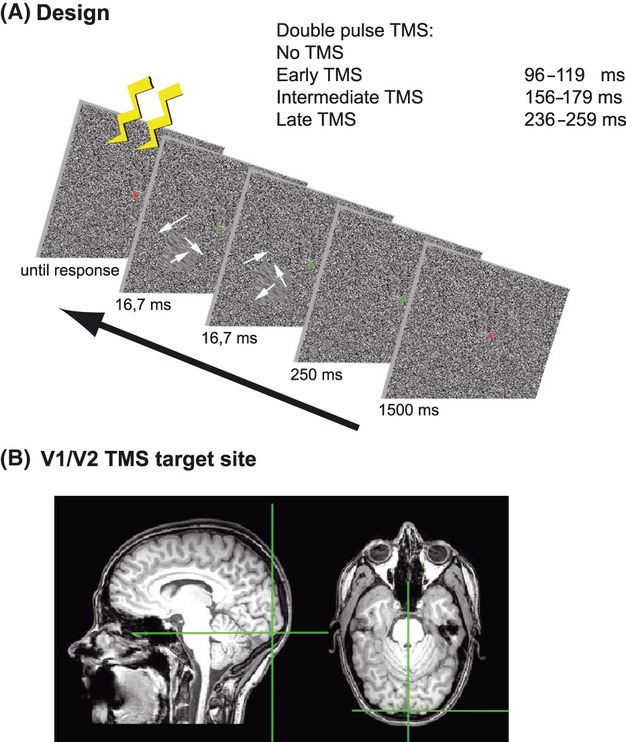
(A) Task design. Participants had to discriminate between a “stack,” “frame,” or “homogenous” stimulus. Crucially, these three stimuli differed in the amount of figure–ground segregation needed to make a correct distinction. The stimulus was presented at the lower left side of the fixation dot. Importantly, after stimulus presentation, we briefly disrupted V1/V2 at various moments after stimulus presentation with two TMS pulses (intermixed with trials without TMS) while recording EEG signals. (B) TMS target location.

Stimuli were presented full screen (1024 × 768 pixels) on a 17-inch DELL TFT (Dallas, TX, USA) monitor with a refresh rate of 60 Hz. The monitor was placed at a distance of ∼90 cm in front of the participant so that each centimeter subtended a visual angle of 0.64°. Participants were instructed to discriminate between a so-called *stack*,*frame*, and *homogenous* stimulus (see Fig. 1A–C). We used stimuli in which figure–ground segregation was achieved by relative motion of random dots. These stimuli were created by placing randomly distributed black-and-white dots (one pixel in size) across the screen. Each pixel had an equal probability of being black or white. A stimulus consisted of three regions: the background (17.99°; 24.8 cd/m²), the figure frame (3.23°; 24.8 cd/m²), and the inner figure (2.42°; 24.8 cd/m²). Stimulus presentation consisted of two screen refreshes (33.3 msec) in which the random dots were displaced one pixel per screen refresh in one of the four directions (45°, 135°, 225°, or 315°). During the first screen, refresh the random dots were displaced in one of the four directions, and during the second screen refresh, the dots were moved one pixel further in that same direction (note that both before and after stimulus presentation, the screen was filled with stationary random dots [for illustration, see Fig. 2A], stimulus presentation merely consisted of moving these dots).

A *homogenous stimulus* was created by displacing the dots of all three stimulus regions coherently in one direction. The *frame stimulus* was created by displacing the dots of the frame region in a different direction than those of the background and inner figure (which were displaced in the same direction), so that a frame appeared to be hovering above and moving in a different direction than the background. The *stack stimulus* appeared when the dots of the inner figure region were displaced in one direction, the dots of the frame region in another direction, and background dots in yet another direction, so that a “stack” of figures appeared to be moving in front of the background.

In all three stimuli, the pixels within each region did not cross their fixed border (Fig. 1A–C). As a consequence, all stimuli produced the same amount of flicker due to (dis)appearing dots. Moreover, on average, all three stimuli contained the exact same strength and directions of motion of dots, so that motion energy was fully balanced between stimuli. Finally, stack and frame stimuli were perfectly balanced with respect to local motion contrast: both stimuli contained an equal amount of borders where motion was in orthogonal directions. The only difference between stack and frame stimuli is in the amount of figure surface that can be perceived: in the frame stimulus, only the (relatively small) frame region segregates from background and in the stack stimulus, both frame and inner figure region segregate. For a subject to correctly discriminate between a homogenous and a figure (stack or frame) stimulus, it is sufficient for the visual system to detect figure borders. However, to discriminate between a stack and a frame stimulus, additional figure–ground segregation (surface segregation) is necessary. Note, however, that the stack and frame stimuli share the same amount of border ownership and only differ in the specific types of border assignments (i.e., for the frame, both borders are owned by the same surface, whereas for the stack, one border is owned by the large occluded square surface and the other by the smaller occluding square surface). We believe that it is highly unlikely that ERPs and TMS are precise enough to measure or disrupt this difference in border assignment. In this study, therefore, it is impossible (and not our intention) to measure or manipulate differences related to border ownership.

Each trial started with a blank screen (1500 msec; 24.8 cd/m²) followed by a display filled with an equal amount of randomly distributed black-and-white dots with a fixation dot placed in the center of the screen (0.15°; 1250–1400 msec, see Fig. 2B). Next, the stimulus (homogenous, frame, or stack) was presented in the lower left corner of the fixation dot (off center: horizontal 7.7°; vertical 10.64°) for two screen refreshes (33.3 msec). After the second displacement, all dots remained in position and the trial ended when a response was given. In the period after stimulus offset, a double TMS pulse could be administered over V1/V2 (see “TMS protocol” below). Participants were instructed to discriminate between the three stimuli and press a left button on a button box placed at the left-hand side (left index finger) when they thought that a homogenous stimulus was presented, press the left button on a button box placed on the right-hand side (right index finger) when they thought a frame was presented, and the right button on a button box placed on the right-hand side (right middle finger) when a stack was presented (target button assignment was counterbalanced across subjects).

The paradigm constituted a 3 × 4 design resulting in 12 trial types, consisting of three different stimuli (stack, frame, and homogenous) and four different TMS conditions (early, intermediate, late, and no TMS, see “TMS protocol”). Within each block, stimulus type and TMS timing were randomized and equally probable. Stimuli were presented using Presentation (Neurobehavioral Systems).

Because of TMS-exposure limitations set by the Ethics Committee, data were gathered in 7–8 sessions per participant (approximately 90 min per session) in which participants performed four experimental blocks per session (25 blocks in total), each containing 96 trials (resulting in 200 trials per condition). All participants were well trained in the experimental task and accustomed to V1/V2 stimulation. Almost all participants (13) already participated in a pilot study (1536 trials) using the same stimuli and almost the same stimulation protocol (single pulse instead of double pulse). Before starting the experimental sessions, all participants received practice trials (four blocks) without TMS. Participants were instructed to keep their eyes fixated on the fixation dot while directing their attention toward the location where the stimuli were presented.

### TMS protocol

We briefly disrupted processing in V1/V2 using a Magstim Rapid² (Magstim Company, U.K.) stimulator. We positioned the base of a 90-mm-diameter circular coil ∼1.5 cm above the inion (central location), with the orientation of the axis of the coil parallel to the transverse plane (handle pointing to the right) and applied a double pulse at 45 Hz (i.e., one pulse followed by another within 23 msec). Current direction was clockwise. We used this location and coil to effectively stimulate areas V1/V2 (considering the anatomical positions of V2 and V1). Participants were placed in a chin rest to optimize stability during stimulation. Before starting the experimental sessions, we determined phosphene threshold as well as the optimal location of the coil, in such a way that the phosphene covered the area where the stimulus would be presented. Before starting the experiment, the phosphene thresholds of each participant were determined by increasing stimulator output while targeting V1/V2 until 50% of the pulses resulted in the perception of a phosphene (eyes open in a dim-lit room, fixating on a black screen). In the experimental setting, we used ∼85% of phosphene threshold to stimulate at three different time intervals (an average of 57% of maximum stimulator output). If participants reported to have seen phosphenes during an experimental block, all data from such an experimental block were discarded. The three TMS time windows were based on behavioral pilot data (25 trials per condition) obtained from four participants (two participants also participated in the TMS–EEG experiment) who were tested at 14 different time intervals (56–339 msec with a 20-msec step) using the same stimuli, coil position, and stimulator settings as during our TMS–EEG experiment (see Fig. S1). We chose three time intervals for our TMS–EEG experiment: an “early” time window (96–119 msec) and a “late” time window (236–259 msec) with a behavioral effect and as a control one “intermediate” interval (156–179 msec) without a behavioral effect. We also presented stimuli without applying TMS (the no TMS condition), creating a total of four TMS conditions (see Fig. 2A).

To rule out any TMS effects unrelated to the disruption of neural activity in V1/V2 (i.e., noisy clicks), we added an extra session in which we applied sham TMS. Seven participants (also participating in the TMS–EEG experiment) performed the discrimination task while sham TMS was applied over V1/V2. We used the same time windows and stimulator output as during actual stimulation. We recorded 48 sham trials per condition (576 trials in total), while an EEG cap was placed on the heads of the participants (although no actual EEG signals were recorded during sham TMS, we wanted to keep the circumstances identical to that of effective stimulation). During sham stimulation, the coil was tilted ventrally, 90° from the plane tangential to the scalp (Lisanby et al. 2001).

### Behavioral analysis

Almost all participants were able to reach a moderate overall performance level. However, two participants failed to reach a level above 67% correct (stack detection remained around chance level). These two participants were excluded so that all further analyses were performed on the remaining 11 participants.

To examine the effect of TMS on behavioral scores, we performed a 3 × 4 repeated measures analysis of variance (ANOVA) on mean percentage correct with factors: stimulus type (homogenous, frame, and stack) and TMS time window (none, early, intermediate, and late). A 3 × 4 repeated measures ANOVA was also performed on mean reaction times (RTs) with factors: stimulus type and TMS time window. RTs of less than 100 and greater than 1500 msec were excluded from all analyses.

### EEG measurements and analyses

EEG was recorded and sampled at 1048 Hz using an ANT 64-channel system with eight bipolar inputs allowing the recording of EOG (ANT – ASA-Lab system of ASA, Enschede, The Netherlands). Sixty-four scalp electrodes were measured, as well as four electrodes for horizontal and vertical eye movements (each referenced to their counterpart). After acquisition, EEG data were filtered using a special filtering algorithm designed to eliminate ringing effects that occur when filtering signals that have high-frequency components. To overcome ringing effects, both the original signal and its mirrored version (transposed in time) are filtered. By combining the filtered original signals and the filtered mirrored signals, it is possible to create epochs around the TMS pulses that are filtered and show no ringing effects (implemented in ASA [ANT – ASA-Lab]). In Matlab (Mathworks, MA, USA), we set EEG sample values to zero in an interval disrupted by the TMS pulses (−2 to 65 msec in relation to TMS onset). Next, we interpolated (using a spline interpolation) the EEG samples set to zero (using data 250 msec before and after the interval set to zero), without affecting EEG samples outside this 67-msec interval (the interpolated segment was of the same order as the rest of the data), so we were able to further filter the data (Sadeh et al. 2011). After initial low-pass filtering (100 Hz) during recording, additional filters were applied after removal of the TMS artifact and data interpolation. High-pass filtering (0.5 Hz), additional low-pass filtering (30 Hz), and a notch filter (50 Hz) were used (doing the filtering before artifact removal would propagate the substantially stronger TMS artifact through the data). To limit the spreading of the interpolated data, we used an infinite impulse response (IIR) filter kernel of limited length. Next, we down-sampled to 256 Hz, and subsequently re-referenced to central medical electrode (Cz). Non-TMS-related artifacts as eye movements were corrected on the basis of independent component analysis (Vigário 1997) and ocular correction (Gratton et al. 1983). Artifact correction was applied on all separate channels by removing segments outside the range of ±75 *μ*V or with a voltage step exceeding 50 *μ*V per sampling point. To increase spatial specificity and to filter out deep sources, we converted the data to spline Laplacian signals (Perrin et al. 1989). After conversion to spline Laplacian signals, trials were manually inspected and removed if irregularities due to interpolation were found. EEG data were baseline corrected by subtracting the average sample value across the 100 msec prior to stimulus presentation. Finally, all trials were averaged per condition. All preprocessing steps were done using Brain Vision Analyzer (BrainProducts, Gilching, Germany), ASA (ANT – ASA-Lab), and Matlab (Mathworks).

We created an a priori pooling of electrodes to increase the signal-to-noise ratio and decrease the amount of comparisons. We based our pooling (O1, O2, Oz, POz, PO3, PO4, PO5, PO6, PO7, and PO8) on previous literature showing neural correlates of figure–ground segregation in these channels (Scholte et al. 2008; Pitts et al. 2011) and where we expected the disruption of TMS would have an effect (Thut et al. 2003).

Although we removed the TMS artifact from our EEG data (see above), the TMS-evoked potential was still present in our data. To cancel out effects in our EEG data related to local dot displacement and the TMS-evoked potential, we subtracted ERPs on trials containing a homogenous stimulus from ERPs on trials containing a figure stimulus (stacks and frames collapsed, see Fig. 5) for each TMS condition separately (Thut et al. 2005; Fahrenfort et al. 2007; Taylor et al. 2007; Sadeh et al. 2011). The resulting difference waves (figure–homogenous difference) now reflect activity related to processing of the figure without activity related to local dot displacement and the TMS-evoked potential. Next, we wanted to study the neural correlate of surface segregation and to cancel out the neural effect of local dot displacement, the TMS-evoked potential and relatively early signals related to figure border processing and border ownership coding. We therefore subtracted ERPs on trials containing frame stimuli from ERPs on stack trials (Fig. 6) for each TMS condition separately. The resulting difference waves (stack–frame difference) now reflect surface segregation and no longer contain activity related to local dot displacement, the TMS-evoked potential, and figure border detection (Scholte et al. 2008).

We performed random-effects analyses by applying sample-by-sample paired *t*-tests (two-tailed) to test which samples of the subtractions differed significantly from zero. We corrected for multiple comparisons by correcting the *P* value by fixing the false discovery rate (FDR) at 0.05 (Benjamini and Hochberg 1995). To reduce the amount of comparisons, we selected time windows that were identified in previous literature (Bach and Meigen 1997; Caputo and Casco 1999; Scholte et al. 2008; Pitts et al. 2011) as relevant for figure border detection, border ownership coding, and surface segregation. We choose a time window between 80 and 230 msec after stimulus onset to statistically test relatively early differences related to figure border detection and border ownership coding (in figure–homogenous subtractions, see above). Note that this time window could not be tested in the condition when TMS was applied in the intermediate time window, due to interpolation of the data (for this condition, all interpolated samples were in the middle of the relevant time window). All interpolated EEG samples were excluded from statistical testing. To study the neural correlates of surface segregation, we choose a time window between 200 and 350 msec after stimulus onset to statistically test differences between ERPs on trials containing stack stimuli and trials containing frame stimuli. Due to data interpolation, we were not able to test this difference in the late TMS condition.

## Results

### Task overview

We constructed a design in which participants had to discriminate between three stimuli. Crucially, these three stimuli differed in type of information needed to make a correct distinction. To discriminate a figure (stack or frame) from a homogenous stimulus, figure border detection is sufficient. However, to discriminate a stack from a frame stimulus (later emerging), surface segregation is essential, as low-level features, figure borders, and the amount of border ownership are equal in both stimuli. With a double TMS pulse, we were able to briefly disrupt neural activity in areas V1/V2 at different moments in time and hence could find out if and when early visual cortex contributes to different stages during figure–ground segregation. By concurrently measuring EEG signals, we were able to investigate the causal role of previously described neural correlates of figure–ground segregation (Lamme et al. 1992; Caputo and Casco 1999; Scholte et al. 2008; Pitts et al. 2011).

### Behavioral data reveal two critical time windows in V1/V2

**Figure 3:**
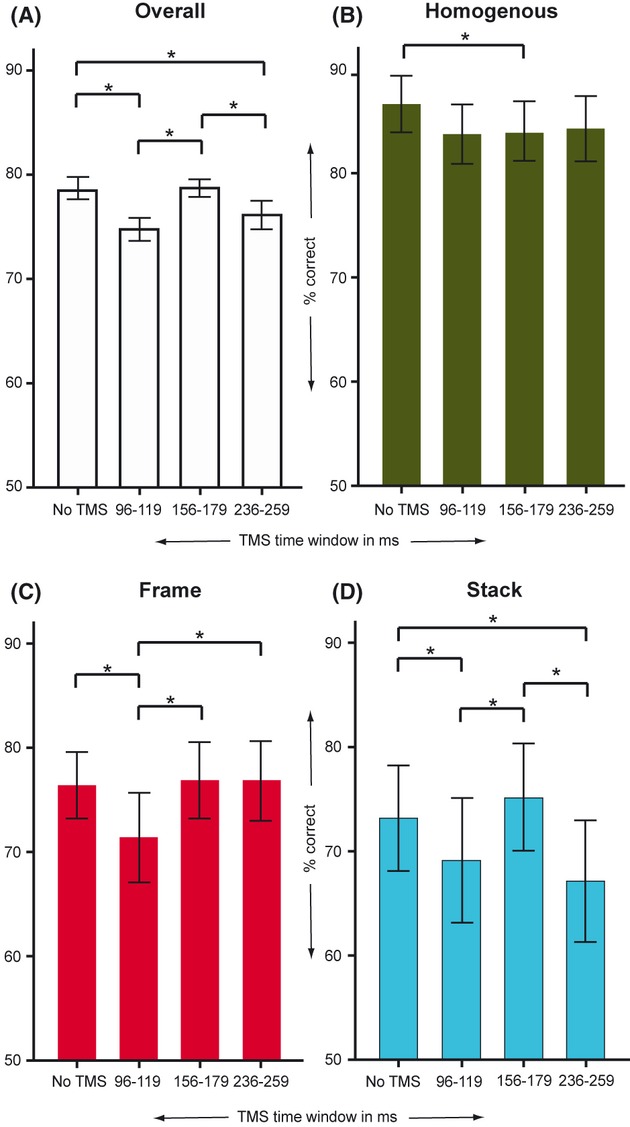
(A) Overall detection scores per transcranial magnetic stimulation (TMS) condition show that performance was affected depending on timing of TMS and stimulus type. (B) TMS in general, not timing specific, seemed to disrupt detection of homogeneous stimuli. (C) Frame detection decreased selectively when TMS was applied in an early time window. (D) Detection of stack stimuli was deteriorated when TMS was applied in this same early time window, but also later in time again in the late TMS condition. Data are means ± SEM.

To test the effect of TMS time window and stimulus type, two 4 × 3 (TMS time window × stimulus type) repeated measures ANOVAs – on accuracy and RTs – were performed. We found a clear interaction between stimulus type and time window of TMS (*F*_(6, 60)_ = 5.30, *P* < 0.001), showing that TMS applied in a specific time window altered performance depending on the type of stimulus presented (Fig. 3B–D). There was a significant main effect of TMS time window on accuracy (*F*_(3, 30)_ = 12.6, *P* < 0.001).

**Figure 4:**
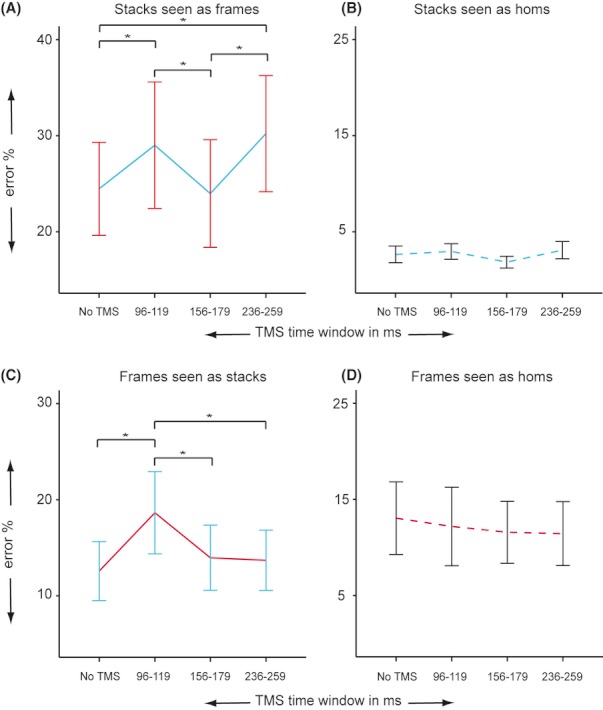
Types of errors are plotted for stacks and frames for the different transcranial magnetic stimulation (TMS) conditions. When TMS was applied in an early time window, stacks and frames are more frequently being mixed up (A and C). When TMS was applied in a late time window selectively stacks are being more often seen as frames (A). TMS has no influence on the amount of stacks seen as homogenous (B) or frames seen as homogenous (D). Data are means ± SEM.

**Figure 5:**
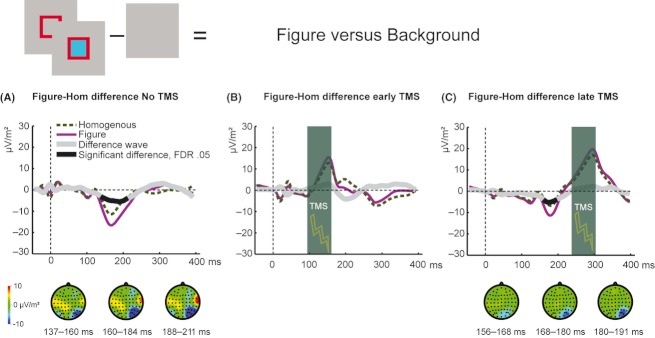
EEG–TMS results: early and late stages in figure–ground segregation. (A) Figure stimuli deflected negatively from homogenous stimuli when no TMS was applied (significant interval = 137–211 msec, *P* < 0.05, corrected for multiple comparison with the FDR) or (C) when TMS was applied in a late time window (significant interval = 156–191 msec, *P* < 0.05, corrected for multiple comparison with the FDR). This significant deflection was abolished when TMS was applied in an early time window (B). Signals behind the transparent vertical bar represent interpolated data. ERPs are computed for a cluster of peri-occipital electrodes (O1, O2, Oz, POz, PO3, PO4, PO5, PO6, PO7, and PO8).

**Figure 6:**
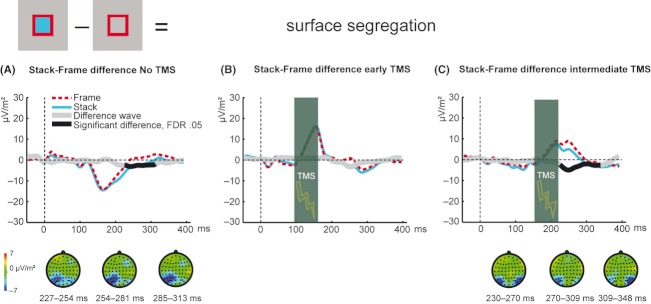
EEG–TMS results: late stage in figure–ground segregation. (A) Stack stimuli significantly deflected from frame stimuli when no TMS was applied (significant interval = 227–313 msec, *P* < 0.05, corrected for multiple comparison with the FDR) or (C) when TMS was applied in an intermediate time window (significant interval = 230–348 msec, *P* < 0.05, corrected for multiple comparison with the FDR). (B) This significant deflection was no longer present when TMS was applied in an early time window. Signals behind the vertical bar represent interpolated data. ERPs are computed for a cluster of peri-occipital electrodes (O1, O2, Oz, POz, PO3, PO4, PO5, PO6, PO7, and PO8). TMS, transcranial magnetic stimulation; EEG, electroencephalography; FDR, false discovery rate; ERP, event-related potential.

Detection of homogenous stimuli deteriorated when TMS was applied, compared with the no TMS condition. This effect was only significant for the intermediate TMS time window (*t*(10) = 2.96, *P* = 0.01, one-tailed, FDR corrected *P* < 0.05). Because there was no difference in detection scores between the three moments when TMS was applied over V1/V2 (i.e., the early, intermediate, and late time windows, all *P*s > 0.66), it thus seemed that, independent of timing, TMS generally caused a slightly elevated tendency to see a homogenous stimulus as a figure, possibly due to a misinterpretation of induced neural noise in V1/V2 (see Fig. 3B).

Frame detection decreased exclusively when TMS was applied in an early time window (Fig. 3C). Performance scores in the early TMS condition were significantly lower in comparison with the no TMS condition (*t*(10) = 2.71, *P* < 0.05, one-tailed, FDR corrected *P* < 0.05), the intermediate TMS condition (*t*(10) = 4.48, *P* < 0.01, one-tailed, FDR corrected *P* < 0.05), and the late TMS condition (*t*(10) = 2.68, *P* < 0.05, one-tailed, FDR corrected, *P* < 0.05).

Stack detection deteriorated when TMS was applied in an early time window (Fig. 3D) in comparison with the no TMS condition (*t*(10) = 2.94, *P* < 0.05; one-tailed, FDR corrected, *P* < 0.05) and the intermediate TMS condition (*t*(10) = 3.83, *P* < 0.01, one-tailed, FDR corrected, *P* < 0.05). Interestingly, Figure 3D shows that applying TMS in the late time window also resulted in poorer detection scores on stack stimuli (no TMS vs. late TMS: *t*(10) = 4.87, *P* < 0.01, one-tailed, FDR corrected, *P* < 0.05; intermediate TMS vs. late TMS: *t*(10) = 5.58, *P* < 0.01, one-tailed, FDR corrected, *P* < 0.05). When we applied TMS in the early time window, performance on both stack and frame stimuli deteriorated, whereas TMS applied in the late time window selectively disrupted detection of stack stimuli.

0Next, we wanted to find out what kinds of errors were being made in the different TMS conditions (see Fig. 4). Analysis of the errors showed that stacks were more seen as frames and vice versa when TMS was applied in an early time window (for frames seen as stacks: early compared with all other TMS conditions, all *t*s(10) >2.38, all *P*s <0.005, two-tailed, FDR corrected, *P* < 0.05; for stacks seen as frames: early vs. no TMS, *t*(10) = 2.30, *P* < 0.05, two-tailed, FDR corrected, *P* < 0.05 and early vs. intermediate TMS, *t*(10) = 2.88, *P* < 0.05, two-tailed, FDR corrected, *P* < 0.05). However, when TMS was applied in a late time window selectively stacks were being more often mistakenly seen as frames (late TMS vs. no TMS, *t*(10) = 3.44, *P* < 0.01, two-tailed, FDR corrected, *P* < 0.05 and late TMS vs. intermediate TMS, *t*(10) = 3.93, *P* < 0.01, two-tailed, FDR corrected, *P* < 0.05).

RT analysis showed no interaction between stimulus type and TMS timing (*F*_(6, 60)_ = 0.59, *P* = 0.75) but did show significant main effects of stimulus type (*F*_(2, 20)_ = 3.95, *P* = 0.04) and TMS timing (*F*_(3, 30)_ = 13.89, *P* < 0.001). Participants responded fastest to homogenous stimuli (mean RT homogenous = 589 msec, SD = 63; mean RT frame = 647 msec, SD = 60; mean RT stack = 614 msec, SD = 73; homogenous vs. frame, *t*(10) = 3.93, *P* < 0.01, two-tailed, FDR corrected, *P* < 0.05) and when no TMS was applied (mean RT no TMS = 588 msec, SD = 43; mean RT early TMS = 620 msec, SD = 60; mean RT intermediate TMS = 621, SD = 57; mean RT late TMS = 639, SD = 59). As we did not find an RT interaction effect of stimulus type and TMS timing, it seems very unlikely that the interaction effect found in performance scores was influenced by a speed–accuracy trade-off. Furthermore, TMS generally (disregarding timing and stimulus type) made participants respond more slowly and less accurate instead of faster and less accurate.

Analysis of data gathered during sham stimulation revealed that our behavioral performance effects were not caused by unspecific TMS effects. No interaction effect (TMS timing × stimulus type, *F*_(6, 36)_ = 0.46, *P* = 0.83) or main effect of TMS timing (*F*_(3, 18)_ = 1.11, *P* = 0.37) was found when we applied sham TMS over early visual cortex. However, analysis of the RTs during sham stimulation showed the same effects as during effective stimulation. We found a significant main effect of stimulus type (*F*_(2, 12)_ = 5.27, *P* = 0.023) and timing of sham stimulation (*F*_(3, 18)_ = 12.81, *P* < 0.001). Post hoc paired *t*-tests showed that participants responded more slowly when sham TMS was applied in comparison with no sham stimulation (no sham separately compared with the three sham conditions, all *t*s(6) >3.39, all *P*s <0.05, one-tailed, FDR corrected, *P* < 0.05). RTs were not influenced by the actual timing of sham stimulation (no difference between sham stimulation in an early, intermediate, and late time window, all *t*s(6) <1.28, all *P*s >0.25). Although our performance scores were not affected by nonspecific TMS effects (unrelated to the disruption of neural activity in V1/V2, such as noisy clicks), it seems that RT differences were mainly driven by unspecific TMS effects.

### Figure versus background

To isolate activity related to figure processing without influences from activity related to local dot displacement and the TMS-evoked potential, we subtracted activity evoked by a homogenous stimulus from activity evoked by a figure stimulus (stack and frame collapsed, see “EEG measurements and analyses”). We first examined the subtraction of these two ERPs without the effect of TMS (Fig. 5A). A difference between figure and homogenous stimuli appeared between 137 and 211 msec (FDR corrected, *P* < 0.05; see “Methods”). When we applied TMS over V1/V2 in an early time window, the significant difference between a figure and a homogenous stimulus is no longer there (Fig. 5B). However, because of the close temporal proximity of the interpolation (see “EEG measurements and analyses”), one should be cautious with interpreting this null result. Not surprisingly (in a causal world), the difference signal was not affected when we applied TMS in the late time window (significant interval of the difference signal: 156–191 msec, FDR corrected, *P* < 0.05; see Fig. 5C). Unfortunately, due to interpolation of the EEG data, we were not able to test the difference between figure and homogenous stimuli when TMS was applied in the intermediate time window (see “EEG measurements and analyses”).

Remarkably, in the no TMS condition, we found a significant deflection between ERPs on figure trials and ERPs on homogenous trials (156–191 msec); however, no behavioral changes were found when TMS was applied during that time window (the intermediate TMS time window, 156–179 msec). Although intuitively this may seem strange, Walsh and Cowey (2000) reported that the peak of the EEG signal does not necessarily have to correspond with the moment when TMS has its behavioral effect. They note that TMS can have a behavioral effect at different moments of the progression of the EEG signal. This difference in timing could be produced by the summative nature of different components in the build-up of the EEG signal, while TMS acts more directly on neural signaling. TMS can thus have an effect when it coincides with the initial build-up of the EEG signal or TMS can have its effect when the EEG signal peaks or even when it is near the end of its decline. In this experiment, it seems that applying TMS in the build-up of the difference between ERPs on trials containing a figure stimulus and trials containing a homogenous stimulus affects performance, whereas stimulating at the peak of this difference in ERPs does not alter performance. This suggests that during build-up, the neural processes leading to figure border detection are more vulnerable to interference than when they have fully evolved.

### Stack versus frame: neural correlates of surface segregation

To isolate signals related to surface segregation and to cancel out signaling related to figure border detection, we subtracted activity evoked by frame stimuli from activity evoked by stack stimuli (as both stimuli have exactly the same amount of figure borders on exact the same locations, see “Task design”). Figure 6A shows a significant deflection between responses evoked by stack and frame stimuli appearing around 230 msec (significant interval: 227–313 msec, FDR corrected, *P* < 0.05) in the no TMS condition. This stack–frame difference was abolished in the early TMS condition (Fig. 6B), where behaviorally stimulation resulted in decreased stack and frame detection. In the intermediate TMS condition, responses evoked by stack and frame stimuli remained to significantly deflect from one another between 230 and 348 msec (FDR corrected, *P* < 0.05; see Fig. 6C). Due to interpolation of the EEG data, we were not able to test the difference between stack and frame stimuli when TMS was applied in the late time window (see “EEG measurements and analyses”).

**Figure 7:**
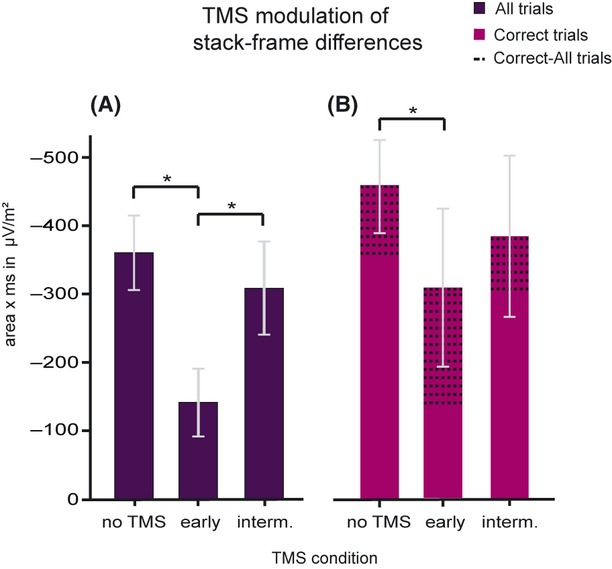
Transcranial magnetic stimulation (TMS) modulation of stack–frame difference. (A) Early TMS reduced the difference in activity evoked by stack and activity evoked by frame stimuli in comparison with the no TMS condition (*t* = 2.97, *P* = 0.01, two-tailed) and the intermediate TMS condition (*t* = 2.50, *P* = 0.04, two-tailed). No difference was found between no TMS and TMS applied in the intermediate time window (*t* = 0.95, *P* = 0.37, two-tailed). (B) Excluding error trials resulted in an increased (trend) stack–frame difference (collapsed across TMS conditions, correct trials vs. all trials: *t* = 1.60, *P* = 0.07, one-tailed). The dashed lines display the increase in the stack–frame difference for each TMS condition after excluding error trials. The stack–frame difference for correct trials is only significant between the no TMS and early TMS conditions (*t* = 2.62, *P* = 0.03, two-tailed). Data are means ± SEM.

TMS stimulation in an early time window decreased figure detection and disrupted relatively early neural signaling associated with figure border detection. In addition, TMS in an early time window disrupted later occurring figure–ground signals related to surface segregation, while neural correlates of surface segregation remained intact when TMS was applied in the intermediate time window. To test whether there is a difference between the different TMS conditions, we compared the difference signals (responses evoked by stack stimuli minus responses evoked by frame stimuli) of three TMS conditions: the no TMS condition, the early TMS condition, and the intermediate TMS condition (the late TMS condition is missing due to data interpolation, see “EEG measurements and analyses”). For each TMS condition, we cumulated values of this difference signal in the time interval between 227 and 313 msec (based on the significant deflection of stack from frame stimuli in the no TMS condition). Figure 7A shows a clear reduction in the difference between ERPs on trials containing a stack and trials containing a frame stimulus when TMS was applied in an early time window in comparison with the no TMS condition (*t* = 2.97, *P* = 0.01, two-tailed) and the intermediate TMS condition (*t* = 2.50, *P* = 0.04, two-tailed). Interestingly, there is no difference between no TMS and TMS applied in an intermediate time window (*t* = 0.95, *P* = 0.37, two-tailed). Next, we explored the relationship between performance (i.e., correctly perceiving a stack as a stack) and late neural signaling in occipital cortex. We expected stack–frame differences to increase by excluding error trials, as these errors trials involved the mix-up of stack and frame stimuli (see Fig. 3). We therefore performed the same analysis as described above (Fig. 7A), but now excluding all error trials. Figure 7B shows that by excluding error trials, we were able to observe an enhancement (trending) of the stack–frame difference (collapsed across TMS conditions, correct-all trials: *t* = 1.60, *P* = 0.07, one-tailed). Comparing different TMS conditions for correct-only trials resulted in a significant difference between the no TMS and early TMS condition (*t* = 2.62, *P* = 0.03, two-tailed). Interestingly, although behaviorally all EEG trials were equal (correct-only trials), we are still able to observe a difference between the different TMS conditions (Fig. 7B). It therefore seems that TMS is able to influence neural signaling, without necessarily leading to overt behavioral effects.

## Discussion

By briefly disrupting activity in early visual cortex during a discrimination task, we were able to causally link activity in areas V1/V2 to different stages in figure–ground segregation. The present findings show that the role of early visual cortex is not limited to low-level computations, but reveal that areas V1/V2 are also necessary later in time when surface segregation emerges. Here, we observed that disruption of V1/V2 activity in the late TMS time window resulted in reduced performance scores selectively for stack stimuli. In order to correctly discriminate a stack stimulus (from a frame stimulus) surface segregation is necessary, therefore causally linking activity in early visual cortex in this relatively late period to surface segregation. In addition, disruption of early visual cortex in this late time window selectively made participants erroneously see more stacks as frame stimuli supporting the claim that specifically surface segregation was affected in this time window (as frames are identical to stack stimuli except for a different amount of figure surface, see “Task design”). The necessity of early visual cortex in this late period during figure–ground segregation demonstrates that late V1/V2 activity is not epiphenomenal or merely a by-product of activity in higher (visual) areas.

Surprisingly, stimulation of early visual cortex in an intermediate time window did not affect performance or neural signaling associated with surface segregation. This latter finding indicates that the degree to which activity in early visual cortex is necessary for figure–ground segregation varies over time.

### The neural pathway of surface segregation

The neural pathway mediating contextual modulations found in nonhuman primates or enhanced ERP components related to surface segregation in early visual cortex has been a topic of debate for many years (Kastner et al. 2000; Lamme and Spekreijse 2000; Rossi et al. 2001; Supèr et al. 2010; Zhang and von der Heydt 2010). Lesion studies (Lamme et al. 1998; Bullier 2001) corroborated by demonstrations on conducting speed of lateral connections (Bringuier et al. 1999; Girard et al. 2001) stress the role of feedback signals in this relatively late phase of figure–ground segregation in early visual cortex. Alternatively, these late processes in early visual cortex could be the product of horizontal connections integrating information over larger parts of the visual field. Local cortical interactions (Das and Gilbert 1999) or long-range horizontal connections (Kapadia et al. 1995) could be dominantly responsible for relaying contextual information within early visual cortex. However, previous studies have demonstrated that the conduction velocity of horizontal connections is ten times as slow as the conduction speed of feedforward or feedback connections (Bringuier et al. 1999; Girard et al. 2001; Angelucci et al. 2002), making integration of information produced by horizontal connections relatively time consuming. The finding of an intermediate period without disruption of neural activity (see Fig. 7) related to surface segregation seems to support the idea that feedback signaling to early visual cortex contributes to this late stage in figure–ground segregation. However, to be able to draw firm conclusions about the role of feedback signals, the inclusion of additional higher tier TMS target locations is necessary.

### Surface segregation and attention

In this experiment, we did not manipulate attention explicitly. Therefore, differences found in our EEG data between stimuli could originate from a difference in amount of attention each stimulus draws (object-based attention, as there is no reason to assume a difference in spatial attention, see “Methods”). Attention modulating activity has been found to travel all the way back to V1 (Roelfsema et al. 1998; Mehta et al. 2000). These modulations by attention seem to enhance processing of relevant regions of a scene while suppressing irrelevant ones (Hopf et al. 2006), thereby shaping visual input for further processing. Considering the temporal aspects of the electrophysiological differences between stack and frame stimuli (>200 msec) in our data, it could be that modulation by attention caused or influenced stack–frame deflections. Recently, however, several studies showed that figure–ground modulation can be found independently from attention (Driver et al. 1992; Kastner et al. 2000; Marcus and Van Essen 2002; Appelbaum et al. 2006; Scholte et al. 2006; but see Ito and Gilbert 1999) and might even guide attentional recourses (Qiu et al. 2007). In this perspective, figure–ground segregation could actually pave the way for prioritizing regions of a visual scene which attention can assign for deeper processing. Thus, if stack–frame differences were caused by attention, these effects would still be depending on the framework generated by figure–ground segregation.

### Form from motion

Classically, it is thought that in stimuli like we used in this study, processing proceeds from motion detection via local motion detectors (e.g., direction selective cells in V1, V3, or middle temporal [MT]), via the detection of motion discontinuities, or motion-defined borders (via opponent motion-sensitive cells, for example, in MT see Movshon et al. 1985; Stoner and Albright 1992) to mechanisms that are selective for motion-defined shapes (e.g., kinetic shape-selective cells in inferior temporal cortex [IT], see Sary et al. 1993). Our experiment was designed so that stack versus frame stimuli were identical with respect to these three stages of processing: on average local motion is identical, stimuli contain the same amount of motion discontinuities, and these motion discontinuities define the same shapes (rectangles). Their only difference lies in the manner in which filling processes proceed over these rectangles: in frame stimuli the interior rectangle is “grouped” with the background, and the frame stands out as a different surface, whereas in stack stimuli the interior rectangle fills in as a separate surface. We find, in accordance with earlier results (Scholte et al. 2008), that these different ways of filling in the rectangles find their neural correlates in relative late cortical processing, the way beyond the timing of the three steps described above. This is probably due to the fact that these three stages are mediated by rapid feedforward sweep processing (Lamme and Roelfsema 2000), whereas the filling in is mediated by re-entrant processing mediated by horizontal and feedback connections. As a consequence, shape (or form) from motion occurs before filling in, yet we designed our stimuli so that from the perspective of motion-defined shape (form from motion) mechanisms, the stack and frame stimuli are comparable, and hence, a form from motion versus no-form from motion differences cannot explain our results.

### Late activity in early visual cortex

When visual information is initially processed by means of feedforward activation, in the right setting, this information can elicit activity throughout the brain, even reaching the highest levels of the cortical hierarchy (Dehaene et al. 2006; Lau and Passingham 2007; Nakamura et al. 2007; van Gaal et al. 2010; Wokke et al. 2011). As visual information travels upstream, neural responses become increasingly more complex. However, it seems that these early visual areas, which are initially important for processing low-level stimulus features, remain necessary during higher level (visual) processes. It has been suggested that after the initial feedforward sweep, early visual cortex acts as an active blackboard (Bullier 2001), available for comparing and integrating outcomes of computations performed higher upstream. In line with this view, Hochstein and Ahissar (2002) argued that feedback signals flowing back to early visual areas are crucial for processing detailed information (“vision with scrutiny”) present in a visual scene (see also Jolij et al. 2011).

Previous studies using TMS revealed the necessity of early visual cortex in a broad range of complex visual processes. For instance, disruption of activity in areas V1/V2 revealed that early visual cortex is essential for feature and conjunction detection (Juan and Walsh 2003), a locus for multisensory interactions (Romei et al. 2007), and necessary during a long time window (outlasting higher tier visual regions) for perceiving natural scenes (Koivisto et al. 2011).

The use of TMS has also proven to be a fruitful way of exposing the importance of interactions between higher and lower (visual) brain regions. For instance, by disrupting activity in relatively higher and lower visual areas, feedback signaling to V1/V2 has been causally linked to visual awareness of motion (Pascual-Leone and Walsh 2001; Silvanto et al. 2005). Recently, the importance of ongoing interactions between different levels of the cortical hierarchy has been described by models of visual processing in which predictive signals about lower level activity flow from higher to lower order cortical regions, while residual error signals, carrying information about the discrepancy between higher level prediction and actual lower level neural activity, flow in the opposite direction (Rao and Ballard 1999). By using a somewhat different task set-up than in this study, TMS might be a useful tool for discriminating neural activity related to the different properties of the predictive coding framework.

For a long time, the nature of activity in early visual cortex correlated with higher level phases during figure–ground segregation has been hotly debated. By using a combination of TMS and EEG, we were able to demonstrate a causal relationship between relatively late activity in early visual cortex (Zipser et al. 1996; Scholte et al. 2008) and surface segregation. The present results are in line with recent findings showing that early visual cortex is involved in a broad range of higher level visual processes, such as perceptual grouping, working memory, and perceptual completion, and early visual cortex has even been associated with the emergence of visual awareness (Roelfsema et al. 1998; Rao and Ballard 1999; Lee and Nguyen 2001; Supèr et al. 2001; Lamme 2003; Ro et al. 2003; Roelfsema et al. 2004; Boyer et al. 2005; Boehler et al. 2008; Harrison and Tong 2009; Brooks and Driver 2010).

## Supporting Information

**Figure S1.** Behavioral results of the pilot experiment. The TMS time windows we used in our main experiment were based on data obtained from a TMS pilot experiment. We tested four participants using 14 different time windows after stimulus presentation (56–339 msec with a 20-msec step) in which we applied a double TMS pulse over V1/V2. We used the same stimuli, coil position, and stimulator settings during the pilot experiment as in the main TMS–EEG experiment. We chose an “early” time window (96–119 msec) and a “late” time window (236–259 msec) with behavioral effect and one “intermediate” time interval (156–179 msec) without a behavioral effect for our main TMS–EEG experiment. Data are means ± SEM.
